# A case of laparoscopically assisted vulvar reconstruction using the gluteal fold flap for anterior enterocele after robot‐assisted radical cystectomy in a woman

**DOI:** 10.1002/iju5.12823

**Published:** 2025-01-02

**Authors:** Yutaro Sasaki, Yasuyo Yamamoto, Makoto Mizuguchi, Saki Kobayashi, Shinji Nagasaka, Takuya Tokunaga, Junya Furukawa

**Affiliations:** ^1^ Department of Urology Tokushima University Graduate School of Biomedical Sciences Tokushima Japan; ^2^ Department of Plastic and Reconstructive Surgery Tokushima University Graduate School of Biomedical Sciences Tokushima Japan; ^3^ Department of Digestive Surgery and Transplantation Tokushima University Graduate School of Biomedical Sciences Tokushima Japan

**Keywords:** anterior enterocele, bladder cancer, cystectomy, gluteal fold flap, laparoscopy, vulvar reconstruction

## Abstract

**Introduction:**

Vaginal complications following radical cystectomy may require surgical treatment. We herein report a case of successful laparoscopically assisted vulvar reconstruction using the gluteal fold flap for anterior enterocele following robot‐assisted radical cystectomy.

**Case presentation:**

A 71‐year‐old Japanese woman underwent robot‐assisted radical cystectomy for bladder cancer (ypT1ypN1M0). The bladder, urethra, bilateral ovaries, and anterior vaginal wall were removed together transvaginally. The Mercedes‐Benz closure technique was performed for vaginal reconstruction using the posterior vaginal wall. Seventeen months after surgery, she complained of a vulvar bulge, and physical examination confirmed a tennis ball‐sized anterior enterocele. Therefore, she underwent laparoscopically assisted vulvar reconstruction using the gluteal fold flap. No recurrence had developed at 6 months postoperatively.

**Conclusion:**

The combination with laparoscopy allows vulvar reconstruction to be performed safely and efficiently.

**Keynote message**

Vaginal complications following radical cystectomy may require surgical treatment. The vulva was reconstructed using the gluteal fold flap for anterior enterocele after robot‐assisted radical cystectomy. Safe and efficient surgery is possible when combined with laparoscopy.

## Introduction

Anterior enterocele is one of the potential postoperative complications of radical cystectomy in women. It not only affects quality of life but can also lead to serious complications such as organ evisceration, necessitating surgical treatment.^1^ We herein report a case of laparoscopically assisted vulvar reconstruction using the gluteal fold flap for anterior enterocele following robot‐assisted radical cystectomy.

## Case presentation

**Fig. 1:**
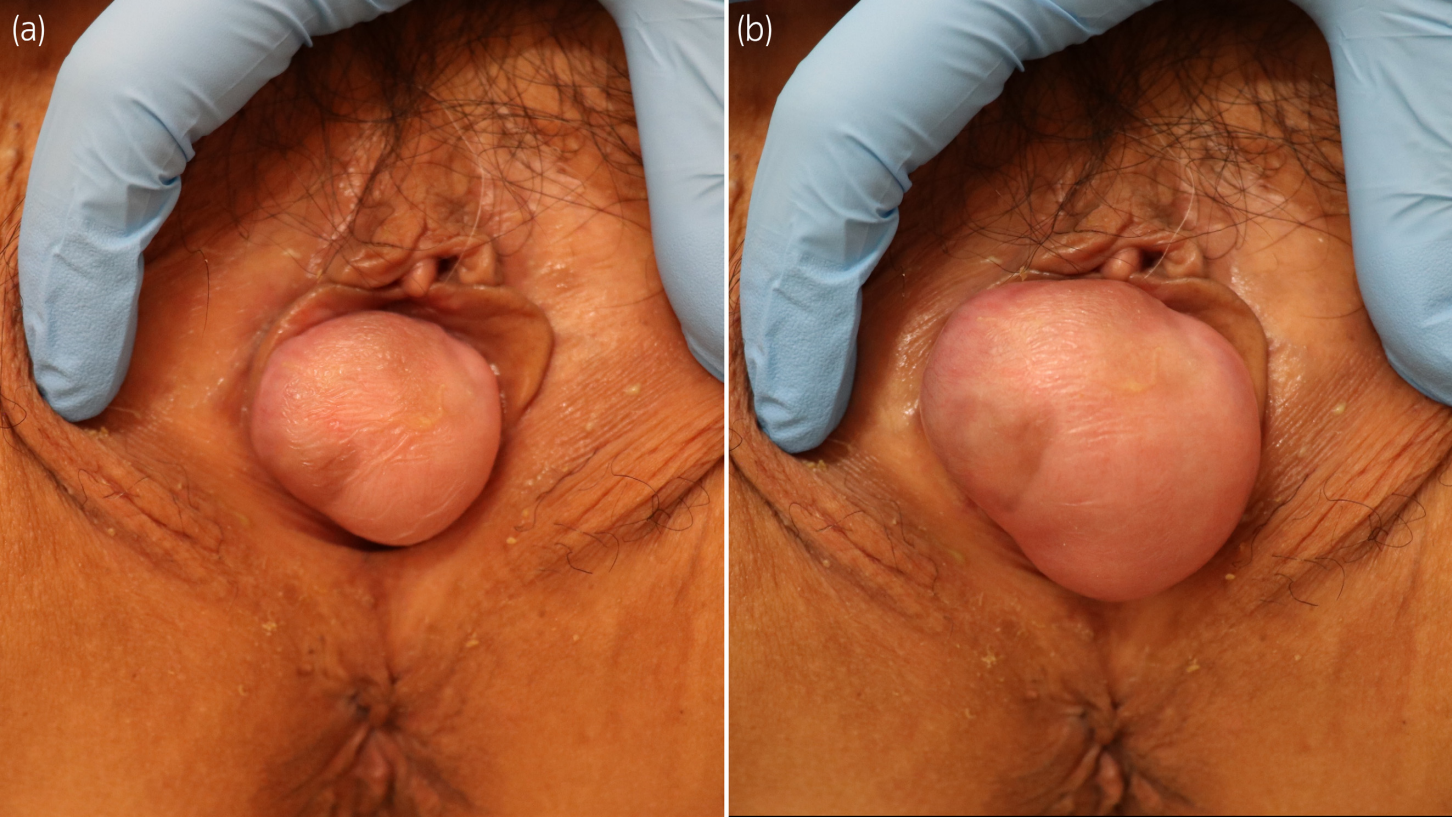
Images showing the tennis ball‐sized anterior enterocele. (a) Image showing the anterior enterocele at rest. (b) Image showing the anterior enterocele during contraction. When intra‐abdominal pressure was applied, the lesion became larger.

**Fig. 2:**
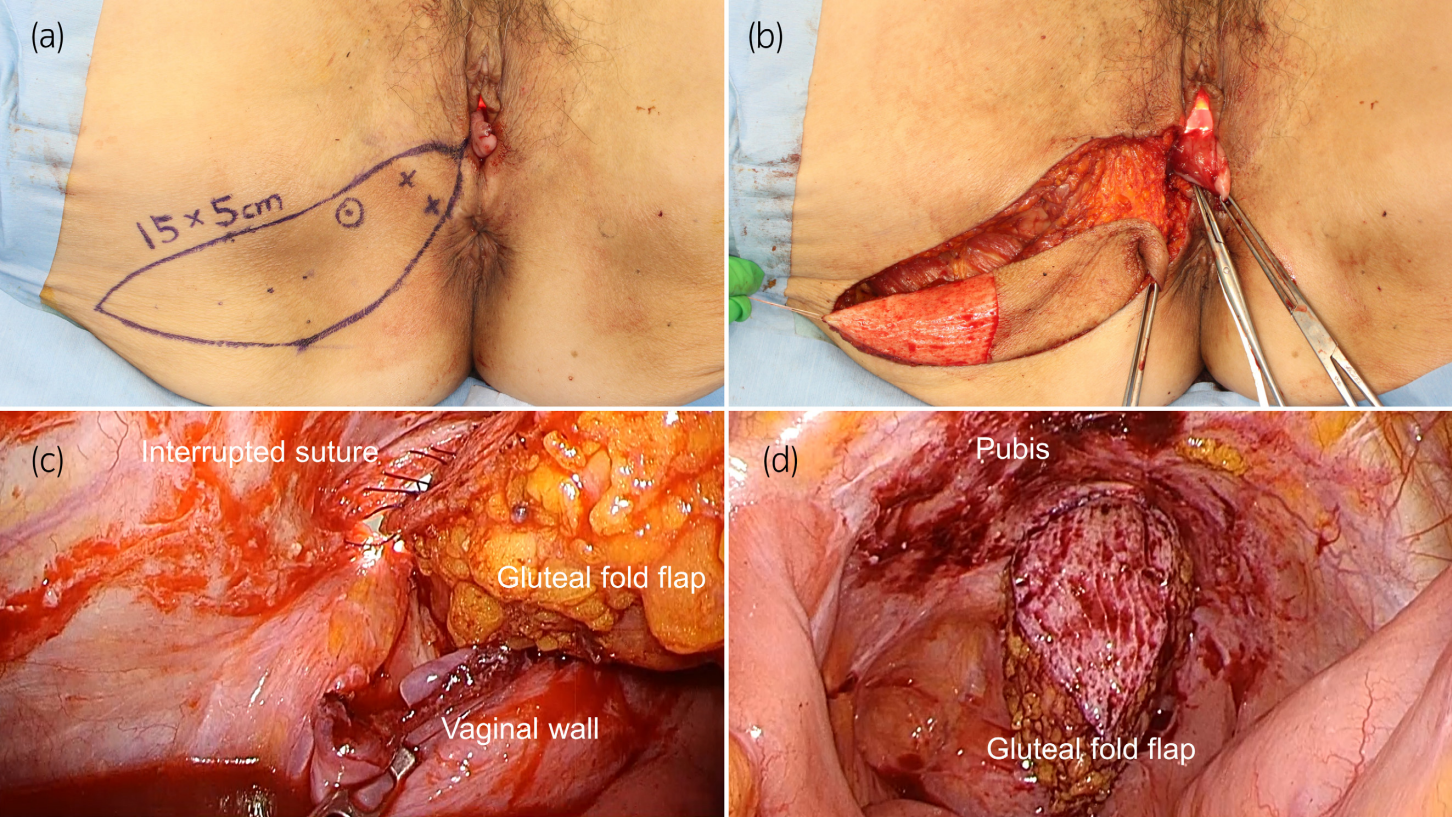
Images showing laparoscopically assisted vulvar reconstruction using the gluteal fold flap. (a) The flap measuring 15 × 5 cm was marked. Internal pudendal artery perforators were identified with a hand‐held Doppler probe (indicated by a circle). (b) The flap was harvested from the right buttock, and its tip was de‐epithelialized. (c) Laparoscopic confirmation was performed to prevent intestinal damage when the flap was inserted into the abdominal cavity and interrupted sutures were placed in the vaginal wall. (d) A laparoscopic image after the flap and vaginal wall were sutured.

**Fig. 3:**
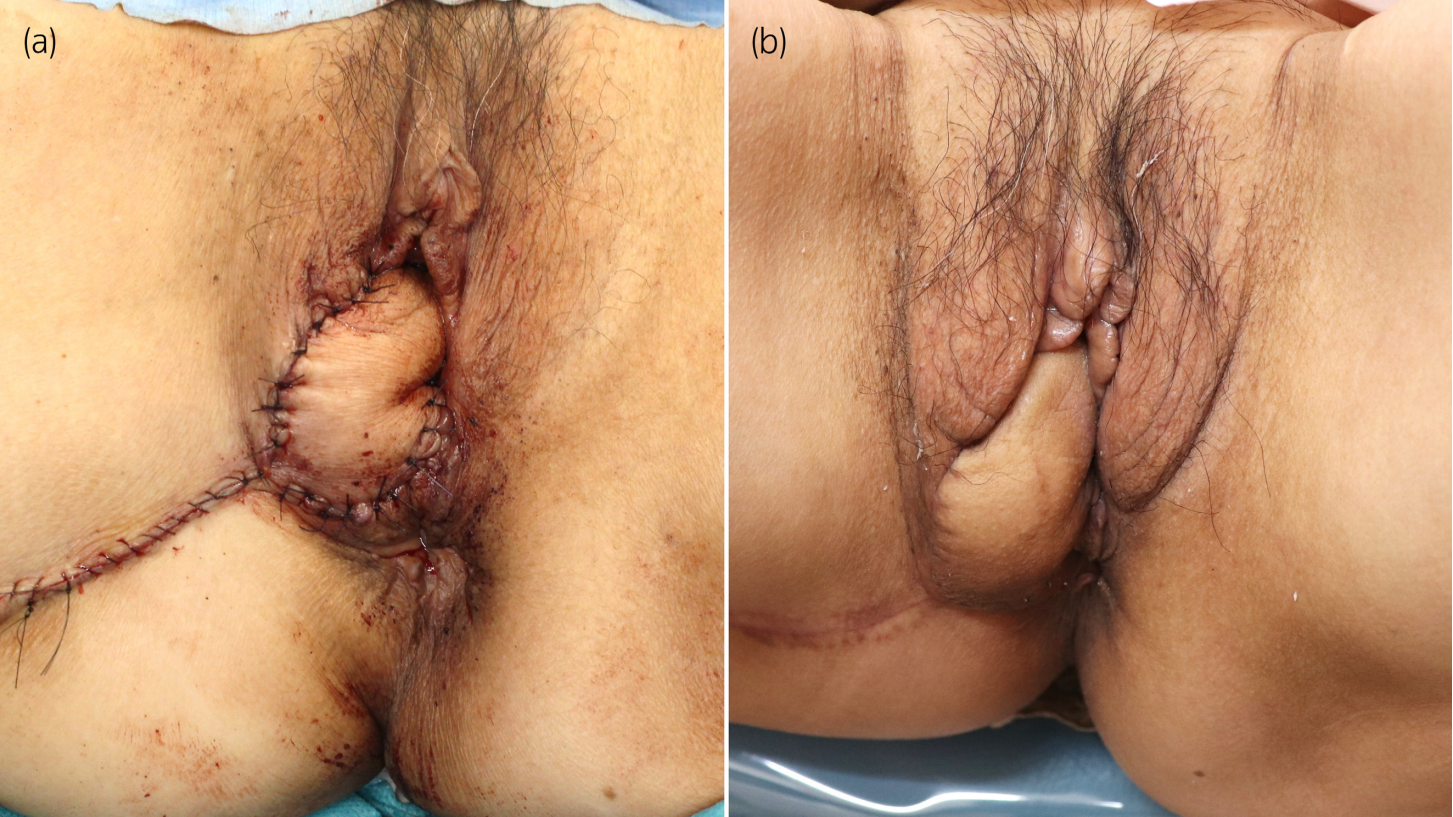
Images showing the vulva after surgery. (a) Image showing the vulva immediately after surgery. (b) Image showing the vulva 6 months after surgery. There was no recurrence of the anterior enterocele.

A 71‐year‐old Japanese woman presented to our institution with complaints of recurrent cystitis. Cystoscopy revealed multiple non‐papillary broad‐based tumors. As a result of transurethral resection of the bladder tumor, she was diagnosed with muscle‐invasive bladder cancer. She had no history of childbirth. Her height, weight, and body mass index were 152.3 cm, 47.0 kg, and 20.3 kg/m^2^, respectively. After neoadjuvant chemotherapy, she underwent robot‐assisted radical cystectomy, pelvic lymph node dissection, and intracorporeal ileal conduit formation for bladder cancer (ypT1ypN1M0). The bladder, urethra, bilateral ovaries, and anterior vaginal wall were removed together transvaginally. The Mercedes‐Benz closure technique was performed for vaginal reconstruction using the posterior vaginal wall.^2^ Although an ileus (grade II of the Clavien‐Dindo classification) developed on postoperative day 2, she was discharged on postoperative day 15. However, 1 year and 5 months after surgery, she complained of a vulvar bulge, and physical examination confirmed a tennis ball‐sized anterior enterocele (Fig. 1). Therefore, she underwent laparoscopically assisted vulvar reconstruction using the gluteal fold flap. The surgical procedure was performed with the patient in the lithotomy position and a 20‐degree Trendelenburg tilt. First, we performed laparoscopic surgery using a 3‐port transperitoneal approach. We performed laparoscopic adhesiolysis of the small intestine that had adhered to the pelvic cavity, and the anterior enterocele was identified. We found that the anterior enterocele had occurred at the vaginal closure site. Next, we performed vulvar reconstruction using the gluteal fold flap. The gluteal fold flap measuring 15 × 5 cm was marked. Internal pudendal artery perforators were identified with a hand‐held Doppler probe (Fig. 2a). The gluteal fold flap was harvested from the right buttock, and its tip was de‐epithelialized (Fig. 2b). The weakened vaginal wall was trimmed. The gluteal fold flap was inserted into the abdominal cavity, and interrupted sutures were placed in the vaginal wall with a braided suture (3‐0 VICRYL RB‐1; Ethicon, Somerville, NJ, USA). At this time, laparoscopy was performed to prevent injury to the small intestine and to properly suture the gluteal fold flap to the vaginal wall (Fig. 2c). Fig. 2d shows a laparoscopic image after the gluteal fold flap was sutured to the vaginal wall, and Fig. 3a shows the appearance immediately after surgery. The operation time was 298 min, and the estimated blood loss was 34 mL. She was placed on bed rest until postoperative day 7 to encourage good flap engraftment. She was discharged on postoperative day 11 without any postoperative complications. No recurrence had developed 6 months postoperatively (Fig. 3b).

## Discussion

Vaginal complications are one of the potential postoperative complications of radical cystectomy in women. Vaginal complications include vaginal prolapse, vaginal fistula, dyspareunia, anterior enterocele, and vaginal cuff dehiscence. Of these, anterior enterocele not only affects quality of life but can also lead to serious complications such as organ evisceration and necessitating surgical treatment.^1^ According to a systematic review of vaginal complications by Richter *et al*., the incidence of vaginal prolapse was 6%–23%.^3^ However, there have been no reports on the incidence of anterior enterocele or the difference in incidence between robot‐assisted and open radical cystectomy. Anterior enterocele requires surgical treatment, such as mesh implants or skin flap surgery. Each surgical treatment has advantages and disadvantages. Mesh implantation is less invasive, but there is a potential risk of mesh‐related complications, including mesh exposure, mesh infection, postoperative pain, and bowel occlusion related to non‐closure of the peritoneum.^4^, ^5^ It also makes repair surgery for recurrent anterior enterocele more difficult. Additionally, there is a lack of data regarding the long‐term outcomes of mesh implants for anterior enterocele following robot‐assisted radical cystectomy. On the other hand, skin flap surgery is more invasive, but we thought it had a higher success rate and fewer complications. Therefore, vulvar reconstruction using the gluteal fold flap was performed by a plastic surgeon. The gluteal fold flap is useful for vulvar reconstruction of perineal defects caused by surgery for vulvar or pelvic malignant tumors.^6^ First, the scar at the donor site is concealed in the gluteal fold, resulting in optimal cosmetic appearance.^6^ Second, the required flap volume can be adjusted, making it useful for a variety of vulvar and buttock reconstructions.^6^ Winterton *et al*. reported on 127 patients who underwent the gluteal fold flap for perineal defects, with a success rate of 97.6%.^7^ In this case, vulvar reconstruction was performed with laparoscopic assistance. We predicted that the small intestine adhering to the pelvic cavity would interfere with the safe and smooth performance of vulvar reconstruction. Therefore, prior to vulvar reconstruction, laparoscopic adhesiolysis was performed. In addition, laparoscopic assistance helped prevent injury to the small intestine and ensured proper suturing of the gluteal fold flap to the vaginal wall. We aimed to avoid any complications in the surgical treatment of anterior enterocele, which is one of the complications of robot‐assisted radical cystectomy. Therefore, the combination of the procedure with laparoscopy allows vulvar reconstruction to be performed safely and efficiently.

## Conclusion

We successfully performed laparoscopically assisted vulvar reconstruction using the gluteal fold flap for anterior enterocele following robot‐assisted radical cystectomy in a woman. We believe that vulvar reconstruction can be safely and efficiently performed using laparoscopy.

## Author contributions

Yutaro Sasaki: Conceptualization; writing – original draft; visualization; investigation. Yasuyo Yamamoto: Conceptualization; writing – review and editing. Makoto Mizuguchi: Investigation; writing – review and editing. Saki Kobayashi: Investigation; writing – review and editing. Shinji Nagasaka: Investigation; writing – review and editing. Takuya Tokunaga: Investigation; writing – review and editing. Junya Furukawa: Conceptualization; investigation; writing – review and editing; supervision.

## Conflict of interest

None.

## Approval of the research protocol by an Institutional Reviewer Board

This research was conducted in accordance with the provisions of the Declaration of Helsinki.

## Informed consent

We obtained consent from the patient's parents for publication of this case report.

## Registry and the Registration No. of the study/trial

Not applicable.

## Animal studies

Not applicable.

## Funding information

This research did not receive any specific grant from funding agencies in the public, commercial, or not‐for‐profit sectors.
